# Comprehensive evaluation of matrix factorization methods for the analysis of DNA microarray gene expression data

**DOI:** 10.1186/1471-2105-12-S13-S8

**Published:** 2011-11-30

**Authors:** Mi Hyeon Kim, Hwa Jeong Seo, Je-Gun Joung, Ju Han Kim

**Affiliations:** 1 Seoul National University Biomedical Informatics (SNUBI), Systems Biomedical Informatics Research Center, and Interdisciplinary Program of Medical Informatics Div. of Biomedical Informatics, Seoul National University College of Medicine, Seoul 110799, Korea; 2 Medical Informatics, Graduate School of Public Health, Gachon University of Medicine and Science, Incheon 40576, Korea; 3 Institute of Endemic Diseases, Seoul National University College of Medicine, Seoul 110799, Korea

## Abstract

**Background:**

Clustering-based methods on gene-expression analysis have been shown to be useful in biomedical applications such as cancer subtype discovery. Among them, Matrix factorization (MF) is advantageous for clustering gene expression patterns from DNA microarray experiments, as it efficiently reduces the dimension of gene expression data. Although several MF methods have been proposed for clustering gene expression patterns, a systematic evaluation has not been reported yet.

**Results:**

Here we evaluated the clustering performance of orthogonal and non-orthogonal MFs by a total of nine measurements for performance in four gene expression datasets and one well-known dataset for clustering. Specifically, we employed a non-orthogonal MF algorithm, BSNMF (Bi-directional Sparse Non-negative Matrix Factorization), that applies bi-directional sparseness constraints superimposed on non-negative constraints, comprising a few dominantly co-expressed genes and samples together. Non-orthogonal MFs tended to show better clustering-quality and prediction-accuracy indices than orthogonal MFs as well as a traditional method, K-means. Moreover, BSNMF showed improved performance in these measurements. Non-orthogonal MFs including BSNMF showed also good performance in the functional enrichment test using Gene Ontology terms and biological pathways.

**Conclusions:**

In conclusion, the clustering performance of orthogonal and non-orthogonal MFs was appropriately evaluated for clustering microarray data by comprehensive measurements. This study showed that non-orthogonal MFs have better performance than orthogonal MFs and *K*-means for clustering microarray data.

## Background

DNA microarray can simultaneously measure the expression levels of thousands of genes. Increasingly, the challenge is to interpret such data to reveal molecular biological processes and the mechanism of human diseases. One of the main goals of expression data analysis is to identify the changing and unchanging genes and to correlate these changes with similar expression profiles. One of the major challenges for gene expression analysis is the reduction of dimension. Gene expression data typically have high dimensionality, with tens of thousands of genes whereas the number of observations or experiments is usually under a hundred. Because the number of variables easily exceeds that of experiments, dimension reduction is obviously required for gene expression analysis. This task can be considered as a matrix factorization problem.

Matrix factorization (MF) methods on microarray data can extract distinct patterns from the data [1-5]. Principal Component Analysis (PCA) and Singular Value Decomposition (SVD) are popular analysis methods, and they have been applied to classification problems with satisfactory results [1,5]. However, because of the holistic nature of PCA or SVD, it is difficult to provide the biologically instinctive interpretation of data from the obtained components. In order to overcome this limitation, Paatero and Tapper [6] and Lee and Seung [7] proposed that non-negative matrix factorization (NMF) can learn part-based representations that can provide the obvious interpretation. The non-negativity constraints make the representation purely additive (allowing no subtractions), in comparison with many other linear representations such as PCA and Independent Component Analysis (ICA) [8]. Their work was applied to signal processing and text mining. Brunet et al. [9] applied NMF to describe the gene expression profiles of all genes in terms of a few number of metagenes in order to derive meaningful biological information from cancer expression datasets. They clustered the samples into distinct subtypes by metagene expression patterns.

The gene expression patterns can be sparsely encoded by metagenes, implying a few significantly co-expressed genes. Several groups have proposed NMF formulation that enforces the sparseness of the decomposition. Li et al. [10] proposed local NMF (LNMF) that has additional constraints to enforce the sparseness in the NMF. Hoyer [11,12] also proposed NMF formulation that can find parts-based representations by explicitly incorporating the concept of sparseness. Wang *et al*. [13] demonstrated Fisher non-negative matrix factorization (FNMF) that learns localized features by imposing Fisher constraints. Gao and Church [14] attempted to control sparseness by penalizing the number of non-zero entries unlike other methods.

Sample-based clustering, however, is not the only concern in microarray data analysis. Gene-based clustering provides informative sets of tightly co-regulated genes. While sample-based clustering relies on metagenes, gene-based clustering relies on meta-samples. The two processes can be viewed as bi-directionally constrained with each other. Good metagene may support good sample-based clusters and vice versa. Optimizing sample- dimension only, sparseness of gene-dimension is relatively decreased when sparseness of sample-dimension is increased. In result, the minimization problem is convex that was subsequently described by others [11,12,14,15] and resulting matrix cannot support gene-based clusters well. Therefore, optimizing both sample and gene dimension together may be appropriated for clustering of microarray data. Here, we employed a novel non-orthogonal MF algorithm, Bi-directional Non-negative Matrix Factorization (BSNMF), with bi-directional sparseness constraints superimposed on non-negative constraints, comprising a few dominantly co-expressed genes and samples together. The bi-directional optimization process may provide quality clustering with improved biological relevance that may not be achieved by applying MFs for each dimension separately.

Many clustering-based methods are developed to transform a large matrix of gene expression levels into a more informative set of which genes are highly possible to share biological properties. Although clustering-based algorithms for microarray data analysis have been extensively studies, most works have not focused on the systematic comparison and validation of clustering results.

Different algorithms tend to lead to different clustering solutions on the same data, while the same algorithm often leads to different results for different parameter settings. Since there is no consensus on choosing among them, the applicable measures should be applied for assessing the quality of a clustering solution in different situations. For example, when the true solution is known and we can compare it to another solution, Minkowski measure [16] or the Jaccard coefficient [17] is applicable. Whereas, when the true solution is not known, there is no agreed-upon method for validating the quality of a suggested solution. Several methods evaluate clustering solutions based on intra-cluster homogeneity or inter-cluster separation [18,19]. Meanwhile, the prediction of the correct number of clusters is a basic problem in unsupervised classification problems. To solve this problem, a number of cluster validity indices, assessing the quality of a clustering partition have been proposed.

In the present paper, we would like to systematically evaluate various MFs applied to gene-expression data analysis. We compare six MFs, including two orthogonal MFs (i.e. PCA and SVD) and four non-orthogonal MFs (i.e. ICA, NMF and NMF with sparseness constraints (SNMF) and BSNMF) and a well-known unsupervised clustering method, K-means algorithm. All were evaluated by seven cluster-evaluation indices. We evaluated them in view of basic three categories: (1) traditional clustering, (2) orthogonal MFs and (3) non-orthogonal MFs. Predictive power and consistency of the methods are evaluated by using adjusted Rand Index and accuracy index when the class labels of data were available. To evaluate the biological relevance of the resulting clusters from different algorithms, we evaluated the significance of the biological enrichment for the clusters by using Gene Ontology (GO) and biological pathway annotations.

## Results

### Evaluation of each clustering-based method

**Figure 1:**
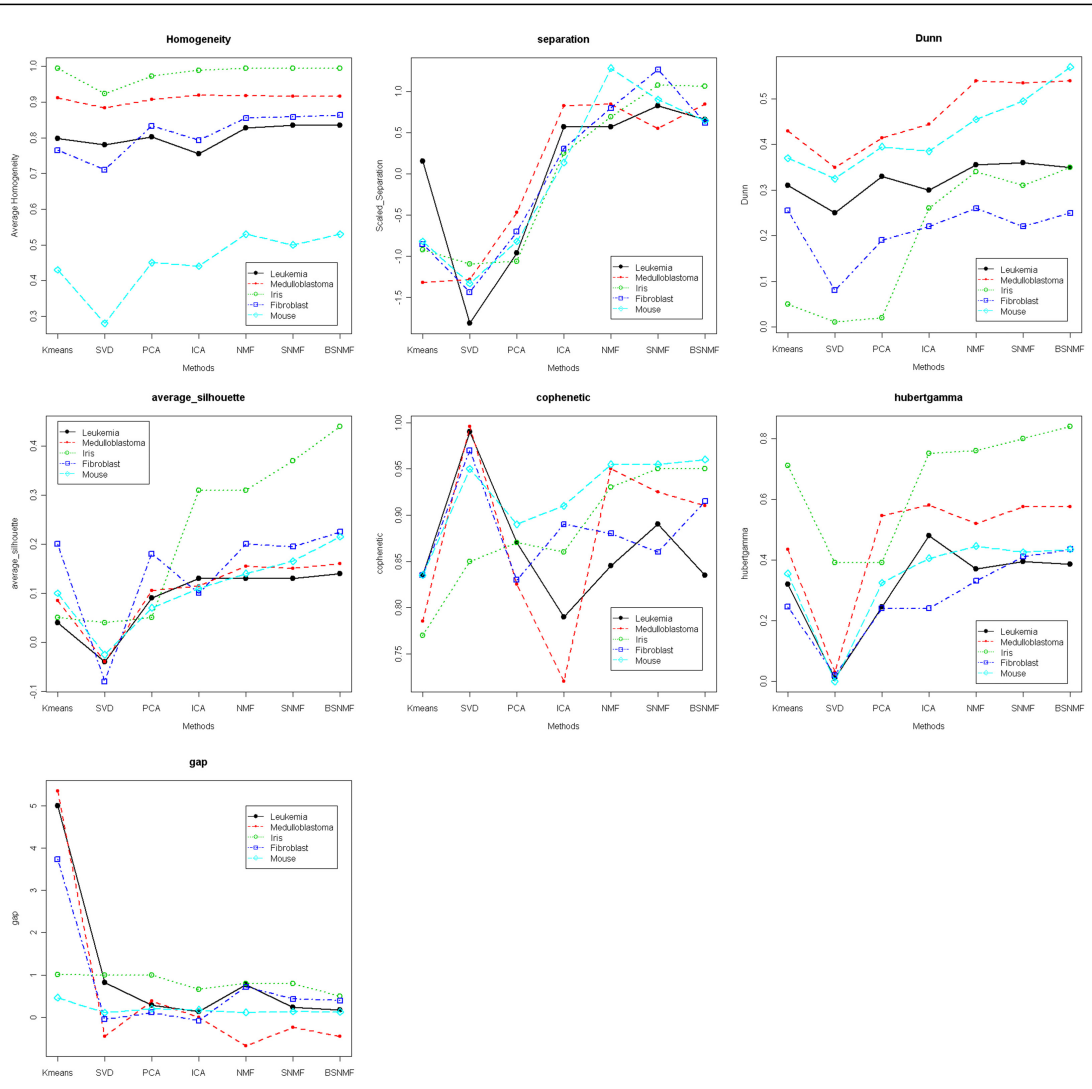
**Illustration of various measures**. Illustration of various measures. Here, we evaluated seven methods by six measures. Each illustration shows results from various measures such as (a) Homogeneity, (b) separation, (c) Dunn Index, (d) average silhouette width, (e) Pearson correlation of cophenetic distance, (f) Hubert gamma and (g) GAP statistic. GAP statistic is optimized when it has lower value. But other measures which have higher value are optimized.

In our study, we applied *K*-means algorithm and six MFs, which are two orthogonal (i.e. SVD and PCA) and four non-orthogonal (i.e. ICA, NMF, SNMF and BSNMF) algorithms to the five benchmarking datasets. We evaluated the seven methods using nine measures, including seven cluster evaluation indices and two prediction power measures. Fig. 1 exhibits results from the seven cluster-quality measures. We repeatedly applied the clustering (or MFs) algorithms 20 times for each dataset for each number of clusters, i.e. *K* = 2 to 4 (for the Iris dataset) or 2 to 5 (for the rest). The values in Fig.1 represent the averages.

Among measures, the GAP statistic is optimized when it decreases (Fig. 1(g)), while others are optimized when they increase (Fig. 1(a) – (f)). The homogeneity, separation, Dunn Index, average silhouette width and Hubert correlation (i.e. Hubert’s gamma) tend to be higher for non-orthogonal MFs than results from orthogonal MFs and *K*-means algorithm. The GAP statistic is lower for non-orthogonal MFs than orthogonal MFs and *K*-means. But, Pearson correlation of cophenetic distance has the highest value for SVD (Fig. 1(e)). Overall, non-orthogonal MFs represented best clustering quality.

We compared homogeneity with separation at the same time (Additional File 1). Results from measures for each dataset were clustered. Results from NMF, SNMF and BSNMF showed higher slope, that is, their homogeneity and separation are more optimized than others. When we compare one of the measures, Hubert correlation of cophenetic distance between MFs, at each number of clusters (Additional File 2), NMF, SNMF and BSNMF showed better performance than others in four datasets except for the Leukemia dataset. ICA has the highest value for the Leukemia dataset. Overall, non-negative MFs have best clustering quality.

**Figure 2:**
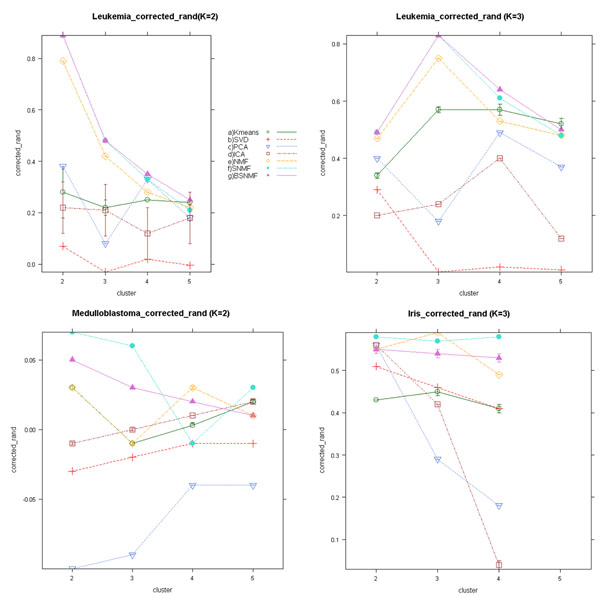
**Illustration of the Adjusted Rand index**. Illustration of the Adjusted Rand index. (a) Result from leukemia dataset which has known class labels with two groups, ALL and AML, We tested various methods at rank *k*=*2*. (b) From leukemia dataset with three groups, ALL-B, ALL-T and AML. We applied the adjusted Rand index at rank *k*=3. (c) From medulloblastoma dataset which has known class labels with two groups, classic and desmoplastic. (d) From iris dataset that has known class labels with three groups of flower species.

The three datasets, Leukemia, Medulloblastoma and Iris datasets have known class labels as ‘gold standards’. For the three datasets, we measured accuracy or predictive power using the adjusted Rand Index and prediction accuracy. Fig. 2 shows the adjusted Rand Index for the correct classification for the three datasets with the seven methods (i.e. six MFs and *K*-means method). The Leukemia dataset was evaluated at both two-class (i.e. AML vs. ALL, Fig. 2(a)) and three-class (i.e. AML vs. T cell type vs. B cell type, Fig. 2(b)) levels. Fig. 2 demonstrates that BSNMF, SNMF and NMF have the highest Adjusted Rand Index for most of the evaluations.

**Figure 3:**
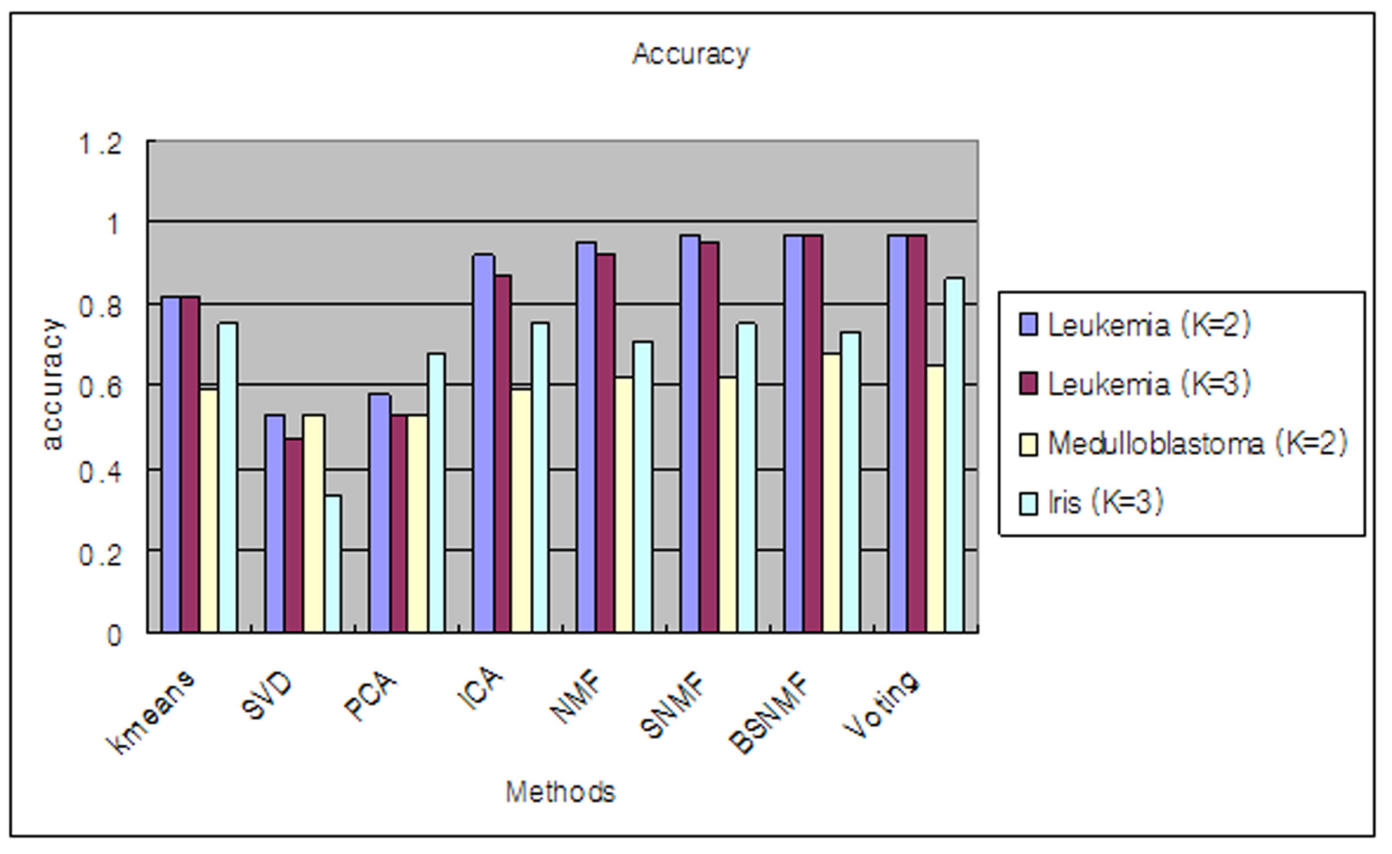
**Illustrations of accuracy.** Illustrations of accuracy. It measures prediction power of clustering. Bar plot of accuracy from three dataset, Leukemia dataset, Medulloblastoma dataset and Iris dataset which have known labels of sample-class.

Fig. 3 shows the results from prediction accuracy. SNMF and BSNMF tend to show the best accuracy measures. We also included a voting scheme that simply combines all the results from the various algorithms and returns the best consensus. Voting showed comparable results to SNMF and BSNMF.

Detailed class prediction results for the Leukemia dataset are shown in Table 1. Class assignment is optimized for each dataset when accuracy has the highest value. All methods were tested both at *K*=2 and *K*=3. At *K*=2 level, one AML sample (AML_12) was incorrectly assigned to ALL by SNMF and BSNMF. The result is the same as that of Gao *et al*. [14]. The error count for NMF was two (ALL_7092_B cell and ALL_14749_B cell). Overall, non-orthogonal MFs like BSNMF, SNMF, NMF and ICA showed higher prediction accuracy than orthogonal MFs and *K*-means algorithm. At *K*=3 level, BSNMF showed the best results with only one mistake that AML_13 was incorrectly assigned to ALL, while SNMF made two mistakes (AML_13 and ALL_21302_B cell). Table 2 shows the results for the Medulloblastoma dataset *K*=2. BSNMF showed the best result with 11 mistakes, while SNMF and NMF have 13 and ICA has 14.

**Table 1:** Class Assignment of Acute Myelogenous Leukemia (AML) and Acute Lymphoblastic Leukemia (ALL)

	Kmeans		^*^SVD		^*^PCA		^*^ICA		^*^NMF		^*^SNMF		^*^BSNMF		^*^Voting	
	(*K*=2)	(*K*=3)	(*K*=2)	(*K*=3)	(*K*=2)	(*K*=3)	(*K*=2)	(*K*=3)	(*K*=2)	(*K*=3)	(*K*=2)	(*K*=3)	(*K*=2)	(*K*=3)	(*K*=2)	(*K*=3)
ALL_19769_B.cell ALL_23953_B.cell ALL_28373_B.cell ALL_9335_B.cell ALL_9692_B.cell ALL_14749_B.cell ALL_17281_B.cell ALL_19183_B.cell ALL_20414_B.cell ALL_21302_B.cell ALL_549_B.cell ALL_17929_B.cell ALL_20185_B.cell ALL_11103_B.cell ALL_18239_B.cell ALL_5982_B.cell ALL_7092_B.cell ALL_R11_B.cell ALL_R23_B.cell ALL_16415_T.cell ALL_19881_T.cell ALL_9186_T.cell ALL_9723_T.cell ALL_17269_T.cell ALL_14402_T.cell ALL_17638_T.cell ALL_22474_T.cell AML_12 AML_13 AML_14 AML_16 AML_20 AML_1 AML_2 AML_3 AML_5 AML_6 AML_7	^**^L L L L L L L L L L L L L L L L L L L L L L L L L L L **L** L ^**^M M **L** **L** **L** M **L** **L** M	^**^B B B B B B B B B B B B B B B B B B B ^**^T T T T T T T T **B** **B** M M **B** **B** **B** M **B** **B** M	**M** L **M** **M** **M** **M** L L **M** L **M** **M** L **M** L L L L **M** L L L L L L L L **L** **L** **L** **L** M M **L** **L** M **L** **L**	B **T** **M** B B B **M** **T** **M** **T** B **M** **M** B **M** **T** **M** **M** B T T T T **M** T T **M** M M M **T** **B** **B** M **T** **B** M **T**	L L L L L L L L L L L L L L L L L L L L **M** **M** **M** L **M** L **M** **L** **L** **L** **L** **L** **L** **L** **L** **L** **L** **L**	**M** B **M** B **M** **M** **M** **M** B B **M** **M** **M** B **M** B **M** **M** B **B** **B** T T T **B** **B** **B** M **B** M M M M M M M M M	L L L L L **M** L L L L L L L L L L **M** L L L L L L L L L L M **L** M M M M M M M M M	B B B B B B B B B B B B B B B B B B B **B** **B** T T T **B** **B** T M **B** M M M M M M M M M	L L L L L **M** L L L L L L L L L L **M** L L L L L L L L L L M M M M M M M M M M M	B B B B B **M** B B B **T** B B B B B B **M** B B T T T T T T T T M M M M M M M M M M M	L L L L L L L L L L L L L L L L L L L L L L L L L L L M **L** M M M M M M M M M	B B B B B B B B B **T** B B B B B B B B B T T T T T T T T M **B** M M M M M M M M M	L L L L L L L L L L L L L L L L L L L L L L L L L L L M **L** M M M M M M M M M	B B B B B B B B B B B B B B B B B B B T T T T T T T T M **B** M M M M M M M M M	L L L L L L L L L L L L L L L L L L L L L L L L L L L M **L** M M M M M M M M M	B B B B B B B B B B B B B B B B B B B T T T T T T T T M **B** M M M M M M M M M

Error Count	7	7	18	20	16	18	3	5	2	3	1	2	1	1	1	1

Class Assignment of Acute Myelogenous Leukemia (AML) and Acute Lymphoblastic Leukemia (ALL) at *K*=2 and *K*=3.

* SVD: singular value decomposition, PCA: principal component analysis, ICA: independent component analysis, NMF: non-negative matrix factorization, SNMF: sparse non-negative matrix factorization,

BSNMF: bi-directional non-negative matrix factorization, Voting: Voting class

** L: ALL, M: AML, B: ALL_B cell, T: ALL_T cell

Bold-faced: misclassified samples

**Table 2:** Class assignment for Medulloblastoma dataset

☐ Sample	Subgroup	Kmeans	^*^SVD	^*^PCA	^*^ICA	^*^NMF	^*^SNMF	^*^BSNMF	^*^Voting
Brain_MD_7 Brain_MD_59 Brain_MD_20 Brain_MD_21 Brain_MD_50 Brain_MD_49 Brain_MD_45 Brain_MD_43 Brain_MD_8 Brain_MD_42 Brain_MD_1 Brain_MD_4 Brain_MD_55 Brain_MD_41 Brain_MD_37 Brain_MD_3 Brain_MD_34 Brain_MD_29 Brain_MD_13 Brain_MD_24 Brain_MD_65 Brain_MD_5 Brain_MD_66 Brain_MD_67 Brain_MD_58 Brain_MD_53 Brain_MD_56 Brain_MD_16 Brain_MD_40 Brain_MD_35 Brain_MD_30 Brain_MD_23 Brain_MD_28 Brain_MD_60	classic classic classic classic classic classic classic classic classic classic classic classic classic classic classic classic classic classic classic classic classic classic classic classic classic desmoplastic desmoplastic desmoplastic desmoplastic desmoplastic desmoplastic desmoplastic desmoplastic desmoplastic	^**^2 ^**^1 1 1 1 **2** 1 1 1 **2** **2** **2** **2** 1 1 **2** **2** 1 **2** **2** 1 1 1 1 **2** 2 2 2 **1** 2 2 2 **1** **1**	**2** 1 1 **2** 1 **2** **2** **2** 1 1 1 1 1 1 **2** **2** **2** **2** 1 **2** 1 1 **2** 1 **2** **1** 2 **1** **1** 2 2 2 **1** 2	**2** 1 1 **2** **2** **2** **2** **2** **2** **2** **2** **2** **2** 1 1 **2** **2** 1 **2** **2** 1 1 1 1 **2** 2 2 2 2 2 2 2 2 2	**2** 1 1 1 **2** **2** 1 1 1 **2** **2** **2** **2** 1 1 **2** **2** 1 **2** **2** 1 1 1 1 **2** 2 2 2 **1** 2 2 2 2 **1**	**2** 1 1 1 1 **2** 1 1 1 **2** **2** **2** **2** 1 1 **2** **2** 1 **2** **2** 1 1 1 1 **2** 2 2 2 **1** 2 2 2 2 **1**	**2** 1 1 1 1 **2** 1 1 1 **2** **2** **2** **2** 1 1 **2** **2** 1 **2** **2** 1 1 1 1 **2** 2 2 2 2 2 2 2 **1** **1**	**2** 1 1 1 1 **2** 1 1 1 **2** **2** **2** **2** 1 1 **2** **2** 1 **2** **2** 1 1 1 1 **2** 2 2 2 2 2 2 2 2 2	**2** 1 1 1 1 **2** 1 1 1 **2** **2** **2** **2** 1 1 **2** **2** 1 **2** **2** 1 1 1 1 **2** 2 2 2 **1** 2 2 2 2 2

Error Count		14	16	16	14	13	13	11	12

Class assignment for Medulloblastoma dataset at *K*=2

* SVD: singular value decomposition, PCA: principal component analysis,

ICA: independent component analysis, NMF: non-negative matrix factorization,

SNMF: sparse non-negative matrix factorization,

BSNMF: bi-directional non-negative matrix factorization, Voting: Voting class

** 1: classic type, 2: desmoplastic type

Bold-faced: misclassified samples

### Evaluation of biological relevance

To evaluate the biological relevance of the clustering results, we created clusters of genes and assigned them to the corresponding sample-wise clusters. For MFs, we clustered genes by using coefficient matrix of genes. For instance, in the Leukemia dataset factorized by NMF at *K*=2, we clustered genes into two groups by using the coefficient matrix of genes, W, from NMF. Given such a factorization, the matrix W is able to be used to determine the gene cluster membership, that is, a gene *i* is placed in a cluster *j* if the *w_ij_* is the largest entry in row *i*. Applying *K*-means algorithm, we clustered genes using original gene expression data matrix. Then, we labelled gene-cluster corresponding to the labels of sample-cluster.

Gene-wise clusters are annotated by GO terms and biological pathways. We measured the significance of GO term (or pathway) assignment by using hyper-geometric distribution. Here we briefly regard each GO term and biological pathway as a term. Table 3 shows the numbers of significantly enriched terms for the corresponding clusters at *p* < 0.05. For the Leukemia dataset, BSNMF (*N*=535) and NMF (*N*=532) have the highest numbers of significantly enriched terms in ALL. BSNMF has the highest numbers in AML (*N*=280) and in total (*N*=815) (Table 3(a)). Table 3(b) shows the results from Medulloblastoma dataset. In cluster 1, BSNMF (*N*=599) and *K*-means (*N*=517) have the most significantly enriched terms. In cluster 2, SVD (*N*=361) and NMF (*N*=335) have the most terms. The total number of significant terms is the biggest with BSNMF (*N*=805). Table 3(c) demonstrates that the fibroblast dataset has the biggest total number of significant terms for BSNMF (*N*=504). Table 3(d) exhibits the result from the mouse dataset. In cluster 1, BSNMF (*N*=690) and SNMF (*N*=686) have the most significantly enriched terms. In cluster 2, ICA (*N*=114) has the most terms. The total number of significant terms is the biggest with BSNMF (*N*=746). Overall, the numbers of significantly enriched terms resulting from non-orthogonal MFs, BSNMF, SNMF, NMF and ICA, are bigger than those of orthogonal MFs and *K*-means algorithm.

**Table 3:** Number of significantly enriched GO terms (or pathways)

(a) Leukemia dataset							
**☐**	**Kmeans**	**SVD**	**PCA**	**ICA**	**NMF**	**SNMF**	**BSNMF**

ALL	480	389	441	453	532	425	535

AML	85	262	223	222	167	266	280

Total	565	651	664	675	699	691	815

(b) Medulloblastoma dataset							

**☐**	**Kmeans**	**SVD**	**PCA**	**ICA**	**NMF**	**SNMF**	**BSNMF**

classic	517	373	467	479	388	456	599

desmoplastic	58	361	226	213	335	208	206

Total	575	734	693	692	723	664	805

(c) Fibroblast dataset							

**☐**	**kmeans**	**SVD**	**PCA**	**ICA**	**NMF**	**SNMF**	**BSNMF**

cluster1	52	45	71	47	57	41	128
cluster2	32	35	68	27	54	48	69
cluster3	48	24	63	61	37	75	50
cluster4	126	38	37	96	108	60	155
cluster5	54	63	60	33	65	68	102

Total	312	205	299	264	321	292	504

(d) Mouse dataset							

**☐**	**kmeans**	**SVD**	**PCA**	**ICA**	**NMF**	**SNMF**	**BSNMF**

cluster1	593	520	294	258	637	686	690
cluster2	27	61	107	114	38	28	56

Total	620	581	401	372	675	714	746

Number of significantly enriched terms at *α*=0.05

**Figure 4:**
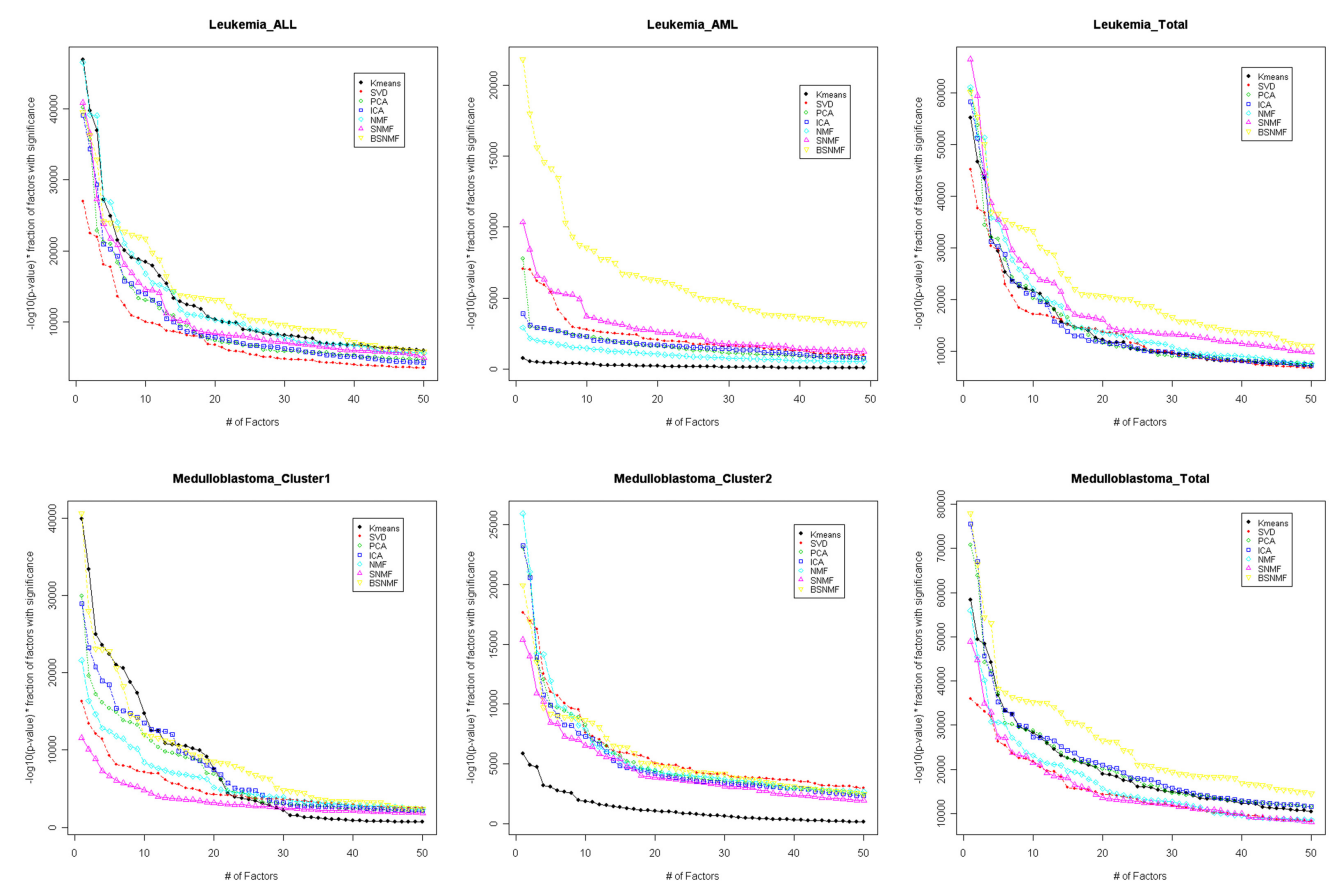
**Weighted p-value of significantly enriched GO terms**. Weighted p-value of significantly enriched GO terms. (a) and (b) represent result of ALL and AML cluster in leukemia dataset. (d) and (e) show result of cluster 1 (assigned to classic type) and cluster 2 (assigned to desmoplastic type) in medulloblastoma dataset. Among the entire significantly enriched factors, top 50 factors are represented. (c) and (f) represent result of top 50 factors in each entire dataset. Results from other dataset are shown in supplementary site.

Dueck *et al*. [20] summarized GO terms with significance to the resulting clusters from various clustering algorithms using two representations: the proportion of factors that are significantly enriched for at least one functional category at *α*=0.05 and the mean log_10_ (*p*-value). We combined two representations. We calculated the weighted *p*-values, the proportion of significant GO terms multiplies the negative log_10_ (*p*-value). Fig. 4 shows the weighted *p*-values of the GO terms significantly annotated to the corresponding clusters for the Leukemia and Medulloblastoma datasets. The weighted *p*-values are more significant when they have higher value. For simplicity, we plotted the top 50 terms. Plots for other dataset can be found in the supplement web site (http://www.snubi.org/software/BSNMF/). For the Leukemia dataset, BSNMF and *K*-means were shown to have annotated terms with the highest significance in AML and BSNMF and SNMF in ALL (Fig. 4(a), (b)). Overall, BSNMF and SNMF showed the highest significance for the whole Leukemia dataset (Fig. 4(c)). In the medullobalstoma dataset, BSNMF and *K*-means for the first cluster and BSNMF and SVD for the second cluster had the higher weighted *p*-value than other methods. Overall, BSNMF showed the best results (Fig. 4(d) - (f)). Therefore, genes in the clusters created by BSNMF seemed to be more biologically associated in terms of GO term annotations than those created by other methods.

**Figure 5:**
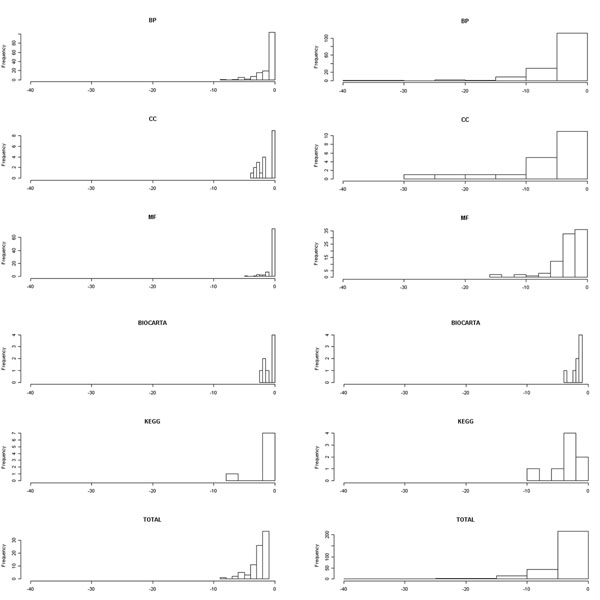
**Log scaled p-values for significantly enriched factors.** Log scaled p-values for significantly enriched factors. Each plot represents significantly enriched terms (at α=0.05) at AML cluster in leukemia dataset using (a) K-means and (b) BSNMF. x-axis represents log10 (p-value). Entire factors were divided into five categories, GO term of biological process (BP), GO term of cellular component (CC), GO term of molecular function (MF), BIOCARTA, and pathway of KEGG.

The *p*-values are calculated for each GO category and for each pathway resource (Fig. 5). The GO term (or pathway) annotation having lower *p*-values represents that corresponding cluster in terms of sharing GO terms (or pathways) is more relevant biologically. The result for *K*-means and BSNMF in the AML cluster is only shown. Other results are found in the supplement web site. Overall, non-orthogonal MFs tend to create more enriched clusters.

The top- ranked genes with the largest coefficient in W matrix of BSNMF may be most explanatory for each cluster (Additional File 3). The top ranked 20 genes for the ALL cluster are enriched in significant GO terms like response to external stimulus, immune response and cell growth. Genes for the AML cluster had are enriched in response to external stimulus, immune response and membrane genes. The gene functions in PubMed indicated that the two sets of 20 genes are enriched in chemokines and tumor suppressor genes. Genes for the first cluster of meduloblastoma were related to cytoplasm, cell motility and cell growth and/or maintenance and those for the second cluster to cytoplasm, biosynthesis and protein metabolism genes. Gene sets for other datasets can be found in the supplement web site.

The mean expression profiles of the gene-wise clusters from the fibroblast dataset were extracted (Additional File 4). We clustered genes by using coefficient matrix of genes when we applied MFs. Coefficient matrix of genes (W matrix) can be used to determine cluster membership of genes, that is, gene *i* belongs to cluster *j* if the *w_ij_* is the largest entry in row *i.* Applying *K*-means algorithm, we clustered genes using original gene expression data matrix. Then, we labelled gene-cluster corresponding to the labels of sample-cluster. According to method mentioned above, gene-wise clusters were created by the seven methods. Number of gene-wise clusters is five because Xu *et al*. [21] and Sharan *et al*. [18] suggested that optimal number of clusters is five from the fibroblast dataset. While *K*-means, SVD and PCA tend to result a few clusters with dominant profiles with the remaining clusters with relatively flat profiles, non-orthogonal MFs tend to create clusters with even dominance. For example, SVD result shows one major peak and BSNMF result shows much more peaks. Non-orthogonal MFs seem to be more effective in discovering significant patterns.

## Discussion

There are various clustering-based methods which are proposed by many researchers. These methods have become a major tool for gene expression data analysis. Different clustering-based methods usually produce different solutions and one or a few preferred solutions among them should be selected. However, a systematic evaluation study for the methods has not been reported. Therefore, we evaluated orthogonal (i.e. PCA, SVD), non-orthogonal (i.e. ICA, NMF and SNMF) MFs and a traditional clustering algorithm (i.e. *K*-means) using seven clustering-quality (i.e. homogeneity, separation, Dunn index, average silhouette width, Pearson correlation of cophenetic distance, Hubert correlation of cophenetic distance and the GAP statistic) and two prediction-accuracy measures (i.e. the adjusted Rand index and prediction accuracy) applying to five published datasets. We also included an improving non-orthogonal MFs, BSNMF in the evaluation study.

As a result, we observed that clustering quality and prediction-accuracy indices applying non-orthogonal MFs are better than those of orthogonal MFs and *K*-means. In respect to results from Homogeneity, separation, Dunn index, average silhouette width and Hubert correlation of cophenetic distance, non-orthogonal MFs had higher value than those of orthogonal MFs and *K*-means. The GAP statistic was lower for non-orthogonal MFs than for orthogonal MFs and *K*-means. When we tested predictive accuracy for the three datasets with known class labels, we also observed better performance for non-orthogonal MFs than for the rest. We also investigated the biological significance of clustering genes because it is important to discover biological relevant patterns and interpret biologically for analysis of DNA microarray gene expression data. When we used enrichment analysis with GO terms and biological pathways, we obtained more significant enriched GO terms or pathways for non-orthogonal MFs than for orthogonal MFs and *K*-means. We also identified genes that may be dominantly involved in each subtype. It was demonstrated that BSNMF showed improved performance in prediction-accuracy and biological-enrichment measures, outperforming other non-orthogonal MFs as well as orthogonal MFs and *K*-means algorithm.

There are various clustering evaluation indices we mentioned. Because they have various results upon datasets, they have limitations to suggest which clustering-based method is the best. Therefore, improving cluster validation indices is needed to overcome it. We simply suggested a voting scheme that simply combines all the results from the various algorithms and returns the best consensus. Improving evaluation indices can be achieved through the integration of results from various evaluation indices using unifying rules.

## Conclusions

In conclusion, the clustering performance of orthogonal and non-orthogonal MFs was appropriately compared for clustering microarray data using various measurements. We clearly showed that non-orthogonal MFs have better performance than orthogonal MFs and *K*-means for clustering microarray data. The characteristic difference among non-orthogonal MFs, orthogonal MFs and *K*-means algorithm implies that non-orthogonal MFs divided whole data into distinct patterns more evenly than orthogonal MFs and *K*-means. This study would help for suitably evaluating diverse clustering methods in other genome-wide data as well as microarray data.

## Methods

### Dataset

For the evaluation study, we used five published datasets. The Leukemia data set [22] has 38 bone marrow samples and 5000 genes after filtering process applied by Brunet *et al.*[9]. Acute myelogenous Leukemia (AML) and acute lymphoblastic leukemia (ALL) are distinguished as well as ALL can be divided into T and B cell subtypes. The second is Medulloblastoma dataset that is a gene expression profiles from the childhood brain tumors. Although the pathogenesis of the tumor is not well understood, it can be categorized into two known histological subclasses: classic and desmoplastic. Pomeroy *et al.*[23] demonstrated the correlation of gene expression profiles and the two histological classes. The dataset has 34 samples and over 5800 genes. The third is the gene expression dataset from Zhang *et al.* (2004, http://hugheslab.med.utoronto.ca/Zhang). This dataset contains gene expression profiles of over 40000 known as well as predicted genes in 55 mouse tissues, organs and cell types. We used over 8200 genes after filtering with low variance. The forth is the human fibroblast gene-expression dataset from Iyer *et al.*[24] with 18 samples and 517 genes. The last is the well-known Iris dataset [25]. This famous dataset gives the measurements in centimetres of the length and width of sepal and petal, respectively, for 50 flowers from each of the three species of Iris (i.e. Iris setosa, versicolor and virginica). Among datasets, Leukemia, Medulloblastoma and Iris dataset have known class labels for samples, while the rest have not.

### Non-orthogonal matrix factorization for gene expression analysis

The gene-expression data is typically represented as an *N*-by-*M* matrix A. In the matrix, each row represents the expression values of a gene across all samples. Each column represents the expression values of all genes in a sample. NMF can decompose gene expression data and derive parts-based representation of the whole data. It factorizes a matrix A into the product of two matrices, including non-negative entries, formulated as A = WH. W and H are *N*-by-*K* and *K*-by-*M* matrices, respectively, and *K* is much smaller than *M*. The column of W can be regarded as a metagene, consisting of elements *w_i_*. Each element represents the coefficient of gene *i* in metagene *j*. The columns of matrix H represent the metagene expression pattern of the corresponding sample. Each element *h_ij_* indicates the expression value of metagene *i* in sample *j*. The cluster membership can be determined based on such a factorization of the matrix H. Sample *j* belongs to cluster *i* if the *h_ij_* is the largest entry in column *j*.

Brunet *et al*. [9] represented parts corresponding to metagenes which represent genes tend to be co-expressed in samples. Here parts mean sets of elements, indicating the building blocks for the whole. These metagenes can overlap, indicating that a single gene may be involved in a number of pathways or biological processes. Therefore, sparseness constraints are needed. NMF with sparseness constraints has been proposed by a few groups. Gao and Church [14] proposed a method to enforce sparseness of H matrix by penalizing the number of non-zero entries. This method enforces sparseness by combining the goal of minimizing reconstruction error with that of sparseness [14]. Specifically, they adopt the point-count regularization approach that enforces sparseness of H by penalizing the number of non-zero entries rather than the sum of entries ∑*h_ij_* in H [11,12,15]. The sparseness is controlled by the parameter and larger parameter makes the H matrix become more and more sparse. Here, the optimization leads the resulting H matrix to contain as many zero entries as possible. Gao’s method enforces sparseness to H matrix only. We applied the sparseness constraints bi-directionally to both W and H. Because microarray gene expression data analysis involves clustering by genes as well as by samples. In microarray data analysis, sample-based clustering can be used to classify samples with similar appearance while gene-based clustering can provide informative sets of tightly co-regulated genes and information about activity of genes. While sample-based clustering relies on metagenes, gene-based clustering relies on meta-samples. The two processes can be viewed as bi-directionally constrained with each other. Good metagene may support good sample-based clusters and vice versa. Optimizing sample-dimension only, sparseness of gene-dimension is relatively decreased when sparseness of sample-dimension is increased. In result, the minimization problem is convex that was subsequently described by others [11,12,14,15] and the resulting matrix cannot support gene-based clusters well. Therefore, optimizing both sample and gene dimension together may be appropriated for clustering of microarray data. This method can optimize both sample and gene clustering. In this paper, we especially focus on BSNMF (Bi-directional Sparseness Non-negative matrix factorization). The definition and algorithm is described below.

**Definition**: Bi-directional Sparseness Non-negative matrix factorization (BSNMF)

Given a non-negative gene expression data V of size *N-*by-*M*, find the non-negative matrices W and H of size *N-*by-*C* and *C-*by-*M* (respectively) such that

E(W, H) = ||V-WH||^2^

is minimized, under optional constraints:

Sparseness (*w_i_*) = λ_1_

Sparseness (*h_i_*) = λ_2_,

where *w_i_* is the *i*^th^ column of W and *h_i_* is the *i*^th^ row of H. Here, *C* denotes the number of components (metagenes), λ_1_ and λ_2_ are the desired sparseness of W and H, respectively. These three parameters are set by the experimenters.

**Algorithm**: Bi-directional Sparseness Non-negative matrix factorization (BSNMF)

1. Initialize W and H to random positive matrices of dimension *N-*by-*C* and *C-*by-*M*, respectively.

2. Rescale the column of W and the row of H to unit norm.

3. Iterate until convergence or reach maximum number of allowed iteration.

(1) If sparseness constraints on H apply

a. solve W_(_*_ia_*_+1)_ = W*_ia_*(VH^T^)*_ia_*/(WHH^T^)*_ia_*

b. Rescale the column of W to unit norm

c. Solve for each *j*

min {1/2||V*_j_*-WH*_j_*||^2^ + 1/2λ_1_||H*_j_*||^2^}

d. if (H*_ij_*<0) then H*_ij_* =0

(2) If sparseness constraints on W apply,

a. solve H_(_*_ia_*_+1)_ = H*_ia_*(W^T^V)*_ia_*/(W^T^WH)*_ia_*

b. Rescale the column of H to unit norm

c. Solve for each *j*

min {1/2||V*_j_*-WH*_j_*||^2^ + 1/2λ_2_||W*_j_*||^2^}

d. if (W*_ij_*<0) then W*_ij_* =0

### Measures of clustering evaluation

In this study, we attempt to evaluate various MFs using cluster evaluation indices. Here, we briefly introduce cluster evaluation indices we used.

***Compactness*** The first measures estimate cluster compactness or homogeneity with intra cluster variance. Many variants of between-cluster homogeneity measure are able to estimate average or maximum pair wise between-cluster distances, average or maximum centroid-based similarities or the use of graph-based approaches [26]. For this purpose, we used the homogeneity index by Sharan and Shamir. **Homogeneity index** is defined as:

In this equation, *C* is a cluster. *C*(*M*) is the cluster centroid and *C*(*x*) is each data item. *Corr*(*C*(*x*), *C*(*M*)) is the correlation coefficient between each data item and the centroid. *N* is the number of data items.

***Separation*** The second index quantifies the degree of separation between individual clusters. For example, the average weighted within-cluster distances define an overall rating for a partitioning, where the distance between individual clusters can be calculated as the distance between cluster centroids, or as the minimum distance between data items belonging to different clusters. Alternatively, we used cluster separation in a partitioning which may be estimated as the minimum separation observed between individual clusters in the partitioning. **Separation** is defined as:

Where *dist*(*C_k_*, *C_l_*) is the minimum distance between a pair of data items, *i* and *j*, with *i* ∈ *C_k_* and *j* ∈ *C_l_*.

***Combinations*** There are a number of enhanced approaches combining measures of the different types of cluster evaluation indices. Several methods therefore estimate both between-cluster homogeneity and within-cluster separation. They compute a resulting score by combining linearly or non-linearly the two measures. A well-known linear combination is the SD-validity Index [27] and non-linear combinations include the Dunn Index [28], Dunn-like-Indices [26], the Davies-Bouldin Index [29] and the silhouette width [30]. We used Dunn Index and average silhouette width. The **Dunn Index** measures the ratio between the smallest cluster distance and the largest between-cluster distance in a partitioning. It is defined as:

where *diam*(*C_m_*) is the maximum intra-cluster distance within cluster *C_m_* and *dist*(*C_k_*,*C_l_*) is the minimum distance between pairs of data items, *i* and *j*, with *i* ∈ *C_k_* and *j* ∈ *C_l_*. The interval of Dunn Index is [0, +∞] and it should be maximized.

The silhouette width for a partitioning is computed as the average silhouette value over all data items [30]. For each observation *i*, the **silhouette width*** s*(*i*) is defined as

where *a*(*i*) is the average dissimilarity between *i* and all other points of the cluster to which *i* belongs. *b*(*i*) is the average dissimilarity of *i* between all observations in its neighbour cluster. A large s(*i*) means that data items are “well clustered”.

***Compliance between partitioning and distance information*** An alternative way of estimating cluster validity is to directly assess the degree to which distance information in the original data is consistent with a partitioning. For that purpose, a partitioning can be represented by means of its cophenetic matrix [31], of which each entry *C*(*i*, *j*) indicates whether the two elements, *i* and *j* are assigned to the same cluster or not. In hierarchical clustering, the cophenetic distance between two observations is defined as the inter-group dissimilarity at which two observations are first joined in the same cluster. The cophenetic matrix can be compared with the original dissimilarity matrix using Hubert’s correlation, the normalized gamma statistic, or a measure of correlation such as the Pearson [32] or Spearman’s rank correlation [33]. We used Hubert’s and **Pearson correlations**. The definition of the **Huber’s correlation** is given by the equation:

where *M* = *N*(*N*-1)/2, *P* is the proximity matrix of the data set and *Q* is an *N*-by-*N* matrix of which (*i*, *j*) element represents the distance between the representative points  of the clusters where the objects *x_i_* and *x_j_* belong.

***Number of clusters*** Most of the internal measures discussed above can be used to assess the number of clusters. If both clustering algorithms employed and the internal measures are satisfactory for the dataset under consideration, the best number of clusters can be obtained by a knee in the resulting performance curve. To measure whether the ‘optimal’ number of clusters is found, we used **Gap Statistic**[34]:

*K* is the total number of clusters giving within dispersion measures *W_k_*, *k* = 1,2,…, *K*. The Gap statistic should be minimized to find the ‘optimal’ number of clusters.

***Predictive power and accuracy*** A number of indices can assess agreement between a partitioning and the gold standard by observing the contingency table of the pair wise assignment of the data items. The well-known index is the Rand Index [35], which determines the similarity between two partitions by penalizing false positive and false negative. There are a number of variations in Rand Index. In particular, the adjusted Rand Index [36] introduces a statistically induced normalization to yield values close to zero for random partitions. Another related indices are the Jaccard coefficient [37] and the Minkowski Score [38]. We used the adjusted Rand Index to estimate the similarity between clustering results and the known class labels. The **Adjusted Rand Index** is defined as:

where *n_lk_* denotes the number of data items assigned to both cluster *l* and cluster *k*. The Adjusted Rand Index has a value in the interval [0, 1] and is to be maximized.

The **accuracy of clustering** is measured by the following formula [39]:

where *I* (*j_i_*) is 1 if the cluster assignment is correct for sample *j_i_*, otherwise 0 if the cluster assignment is incorrect.

### Biological enrichment analysis

We applied biological enrichment analysis to clustering results in order to assess whether functionally related genes are grouped. The resulting genes from clustering are then subdivided into functional categories for biological interpretation. Such functional categorization was accomplished using GO terms and biological pathways. We used DAVID 2.1 (http://david.abcc.ncifcrf.gov/) for GO term enrichment analysis and ArrayXPath [40,41] for pathway annotation. A modified Fisher’s exact test is performed to determine whether the proportions of members falling into each category differ by group, when those in two independent groups fell into one of the two mutually exclusive categories. Therefore, lower *p*-value indicates a better association of cluster members.

## Authors' contributions

MHK conceived of the study, implemented algorithms. HJS revised the manuscript and improved the methodic understanding. JGJ improved the manuscript writing. JHK supervised the study.

## Competing interests

The authors declare that they have no competing interests.

## Supplementary Material

Additional file 1 **Illustration of separation vs. homogeneity** Illustration of separation vs. homogeneity. Results from each dataset are gathered. Each color means each method. Results from NMF, SNMF and BSNMF have higher slope. That is, homogeneity and separation are more optimized. Click here for file

Additional file 2 **Illustration of Hubert gamma** Illustration of Hubert gamma. It is a measure of compliance between partitioning and distance information. Each plot shows result from each datasets at rank *K*=2, 3, 4 (for Iris dataset) or *K*=2, 3, 4 and 5 (for the rest). (a) Leukemia dataset (b) medulloblastoma dataset (c) Iris dataset (d) fibroblast dataset (e) Mouse dataset. Click here for file

Additional file 3 **The twenty common genes in each leukemia subtype** The twenty common genes in each leukemia subtype Click here for file

Additional file 4 **Patterns of mean expression level for each cluster for fibroblast dataset** Patterns of mean expression level for each cluster for fibroblast dataset. (a) *K*-means, (b) SVD, (c) PCA, (d) ICA, (e) NMF, (f) SNMF and (g) BSNMF. Each lines represent for each cluster. Click here for file
