# Supplementary material for: Comprehensive evaluation of matrix factorization methods for the analysis of DNA microarray gene expression data
Source: BMC Bioinformatics. 2011 Nov 30;12(Suppl 13):S8. doi: 10.1186/1471-2105-12-S13-S8 (PMC3278848; doi:10.1186/1471-2105-12-S13-S8)
Supplement: Additional file 1 — Illustration of separation vs. homogeneity Illustration of separation vs. homogeneity. Results from each dataset are gathered. Each color means each method. Results from NMF, SNMF and BSNMF have higher slope. That is, homogeneity and separation are more optimized. [file 1471-2105-12-S13-S8-S1.docx]

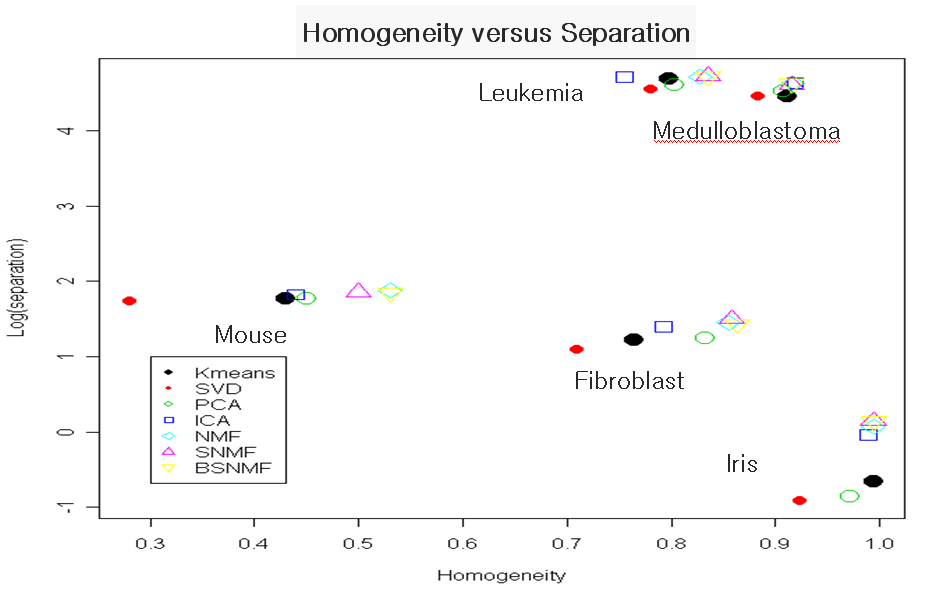


**Supplementary Fig 1.** Illustration of separation vs. homogeneity. Results from each dataset are gathered. Each color means each method. Results from NMF, SNMF and BSNMF have higher slope. That is, homogeneity and separation are more optimized.
