# Supplementary material for: Comprehensive evaluation of matrix factorization methods for the analysis of DNA microarray gene expression data
Source: BMC Bioinformatics. 2011 Nov 30;12(Suppl 13):S8. doi: 10.1186/1471-2105-12-S13-S8 (PMC3278848; doi:10.1186/1471-2105-12-S13-S8)
Supplement: Additional file 2 — Illustration of Hubert gamma Illustration of Hubert gamma. It is a measure of compliance between partitioning and distance information. Each plot shows result from each datasets at rank K=2, 3, 4 (for Iris dataset) or K=2, 3, 4 and 5 (for the rest). (a) Leukemia dataset (b) medulloblastoma dataset (c) Iris dataset (d) fibroblast dataset (e) Mouse dataset. [file 1471-2105-12-S13-S8-S2.docx]

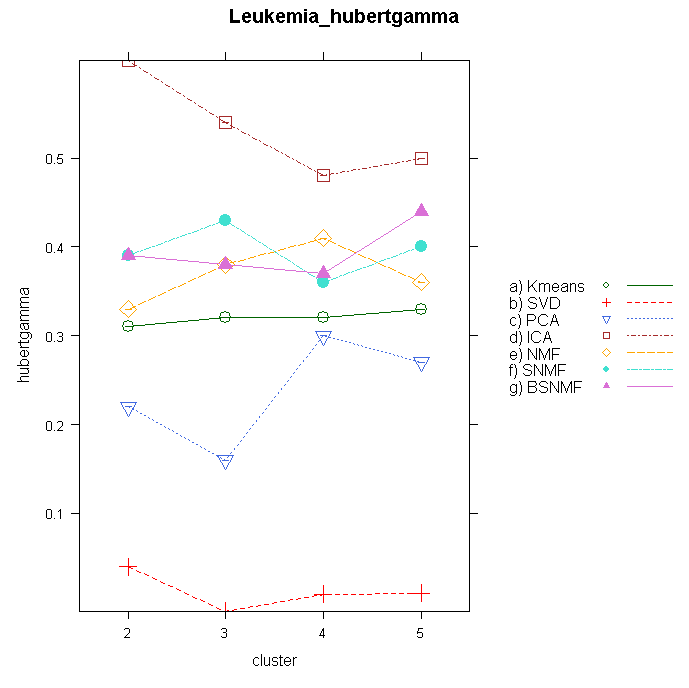

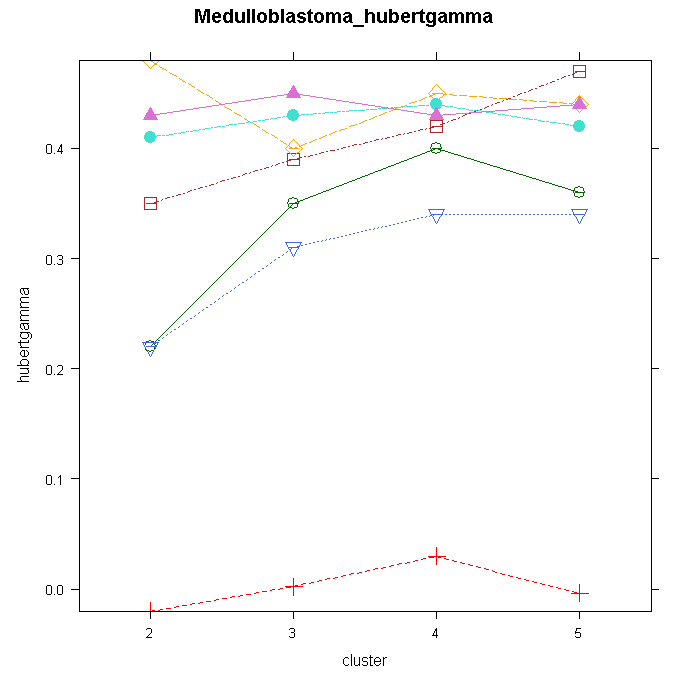


(a) (b)


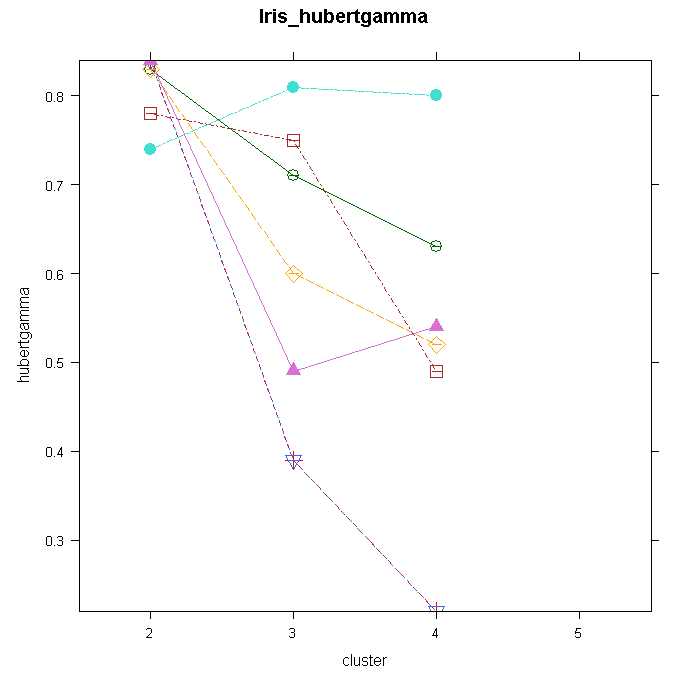


(c)


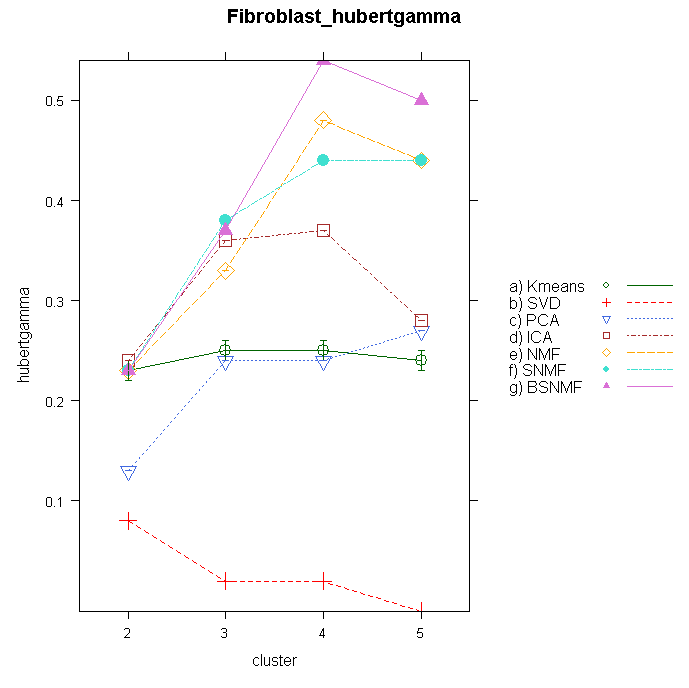

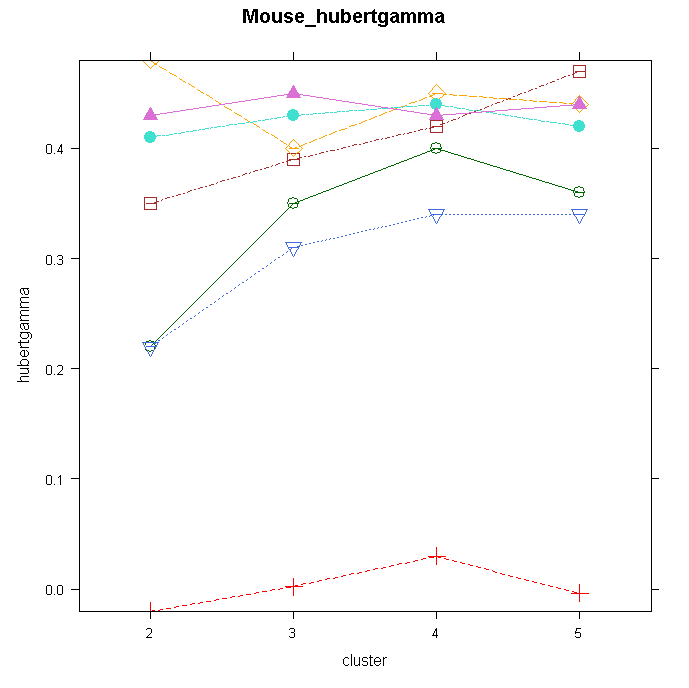


(d) (e)

**Supplementary Fig. 2** Illustration of Hubert gamma. It is a measure of compliance between partitioning and distance information. Each plot shows result from each datasets at rank *K=*2, 3, 4 (for Iris dataset) or *K*=2, 3, 4 and 5 (for the rest). (a) Leukemia dataset (b) medulloblastoma dataset (c) Iris dataset (d) fibroblast dataset (e) Mouse dataset.
