# Supplementary material for: Comprehensive evaluation of matrix factorization methods for the analysis of DNA microarray gene expression data
Source: BMC Bioinformatics. 2011 Nov 30;12(Suppl 13):S8. doi: 10.1186/1471-2105-12-S13-S8 (PMC3278848; doi:10.1186/1471-2105-12-S13-S8)
Supplement: Additional file 3 — The twenty common genes in each leukemia subtype The twenty common genes in each leukemia subtype [file 1471-2105-12-S13-S8-S3.docx]

**Supplementary Table 1. The twenty common genes in each leukemia subtype.**

|  | **Gene Name** |
| --- | --- |
| ^*^ALL | hemoglobin, beta |
|  | CD74 antigen |
|  | immunoglobulin heavy constant mu |
|  | CD81 antigen (target of antiproliferative antibody 1) |
|  | calmodulin 1 (phosphorylase kinase, delta) |
|  | hemoglobin, alpha 1 |
|  | chemokine (C-X-C motif) receptor 4 |
|  | lactate dehydrogenase B |
|  | CD99 antigen |
|  | dual specificity phosphatase 1 |
|  | major histocompatibility complex, class II, DR beta 1 |
|  | cytochrome b-245, alpha polypeptide |
|  | interferon induced transmembrane protein 3 (1-8U) |
|  | heterogeneous nuclear ribonucleoprotein A1 |
|  | Lysosomal-associated multispanning membrane protein-5 |
|  | adenosine deaminase |
|  | thioredoxin interacting protein |
|  | major histocompatibility complex, class II, DR alpha |
|  | T cell receptor beta locus |
| ^*^AML | H1 histone family, member X |
|  | lysozyme (renal amyloidosis) |
|  | glutathione peroxidase 1 |
|  | major histocompatibility complex, class I, E |
|  | S100 calcium binding protein A9 (calgranulin B) |
|  | histone 2, H2aa |
|  | superoxide dismutase 2, mitochondrial |
|  | interleukin 1, beta |
|  | ferritin, light polypeptide |
|  | proteoglycan 1, secretory granule |
|  | interleukin 8 |
|  | metallothionein 2A |
|  | ferritin, heavy polypeptide 1 |
|  | immunoglobulin kappa constant |
|  | cystatin SA |

* ALL: ALL cluster, AML: AML cluster
