# Supplementary material for: Comprehensive evaluation of matrix factorization methods for the analysis of DNA microarray gene expression data
Source: BMC Bioinformatics. 2011 Nov 30;12(Suppl 13):S8. doi: 10.1186/1471-2105-12-S13-S8 (PMC3278848; doi:10.1186/1471-2105-12-S13-S8)
Supplement: Additional file 4 — Patterns of mean expression level for each cluster for fibroblast dataset Patterns of mean expression level for each cluster for fibroblast dataset. (a) K-means, (b) SVD, (c) PCA, (d) ICA, (e) NMF, (f) SNMF and (g) BSNMF. Each lines represent for each cluster. [file 1471-2105-12-S13-S8-S4.docx]

**
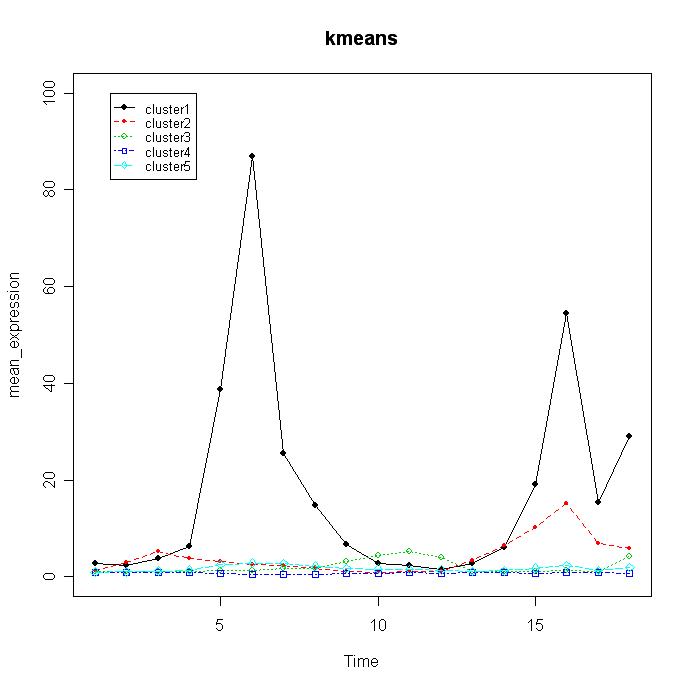

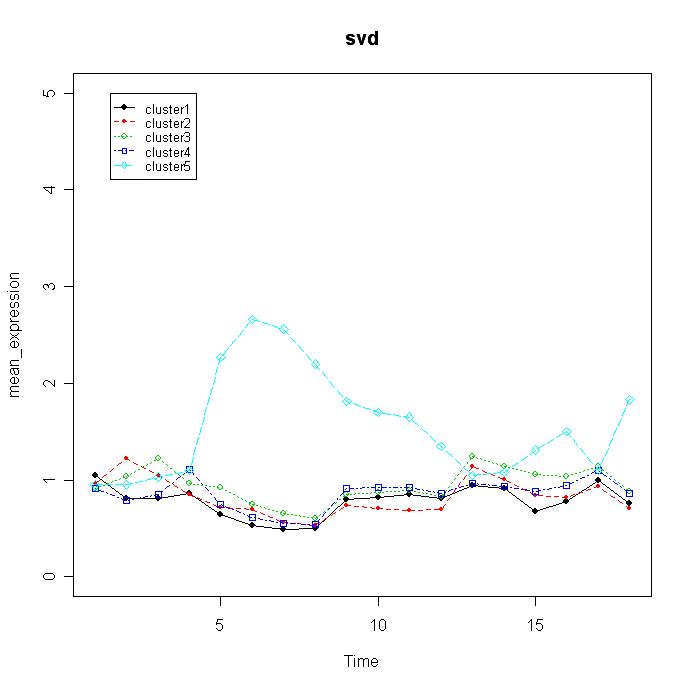
**

(a) (b)

**
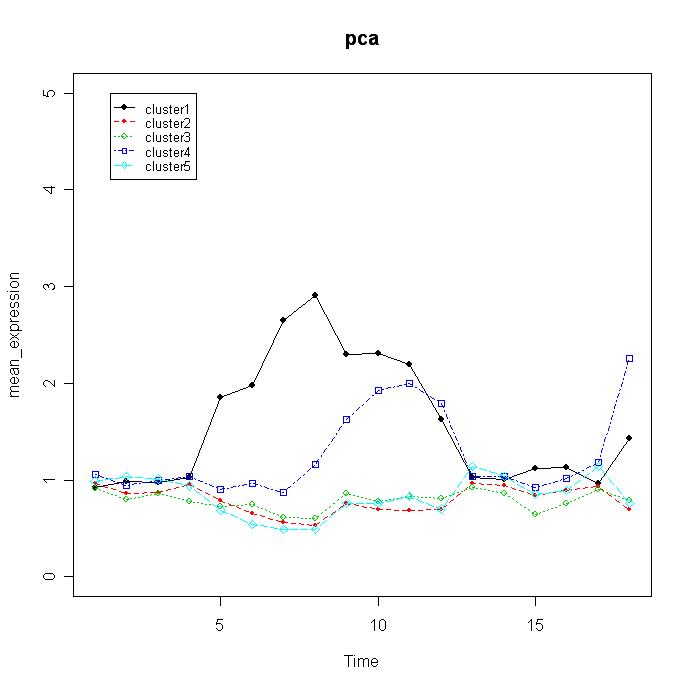

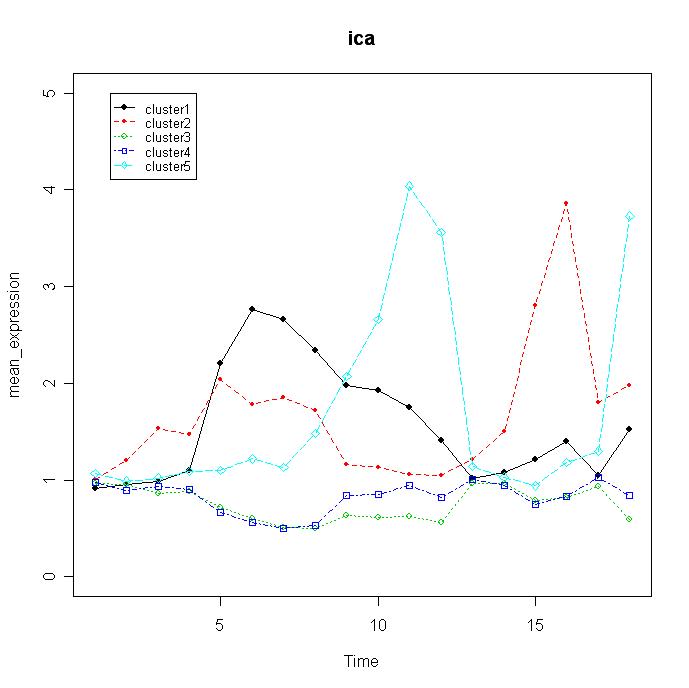
**

(c) (d)

**
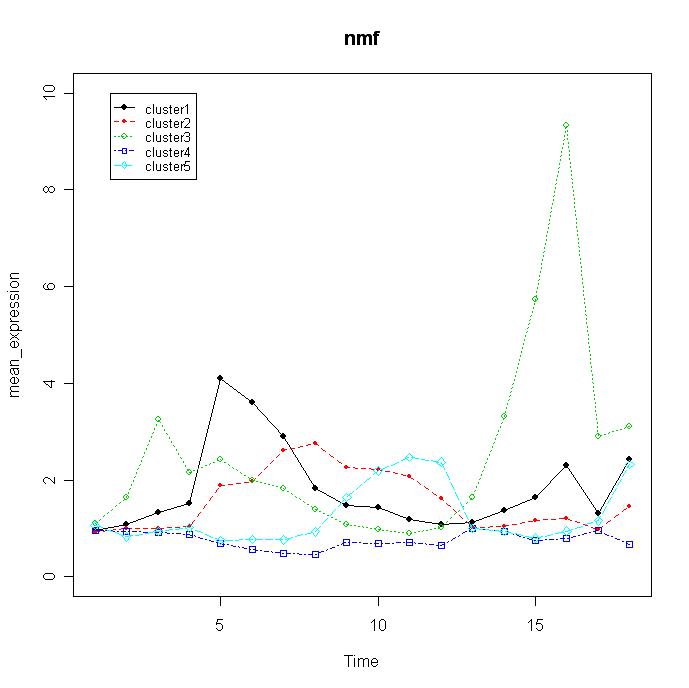

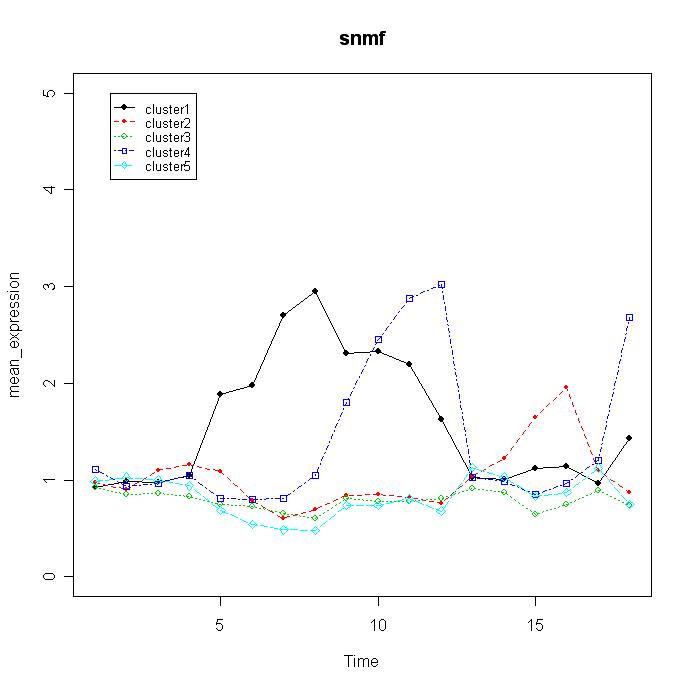
**

(e) (f)

**
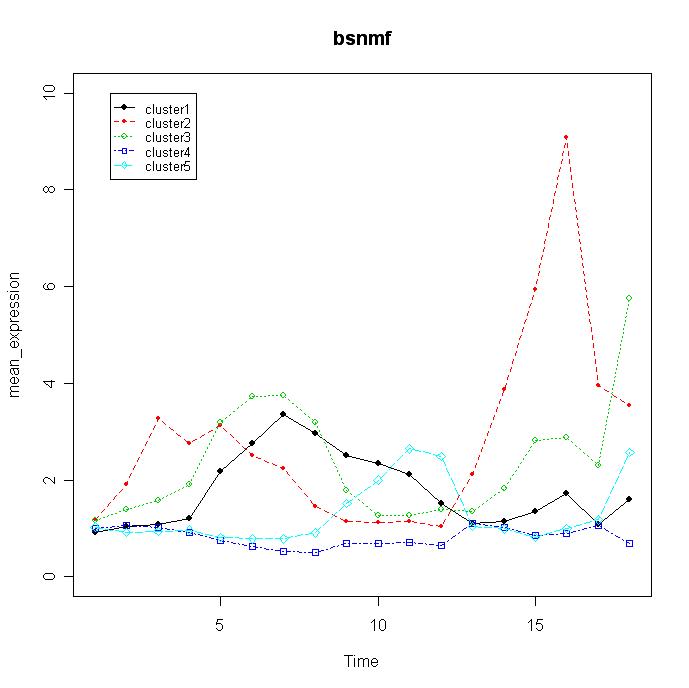
**

(g)

**Supplementary Fig. 4** Patterns of mean expression level for each cluster for fibroblast dataset. (a) *K*-means, (b) SVD, (c) PCA, (d) ICA, (e) NMF, (f) SNMF and (g) BSNMF. Each lines represent for each cluster.
